# Medical knowledge and teamwork predict the quality of case summary statements as an indicator of clinical reasoning in undergraduate medical students

**DOI:** 10.3205/zma001291

**Published:** 2019-11-15

**Authors:** Sophie Fürstenberg, Viktor Oubaid, Pascal O. Berberat, Martina Kadmon, Sigrid Harendza

**Affiliations:** 1 University Medical Center Hamburg-Eppendorf, III. Department of Internal Medicine, Hamburg Germany; 2 German Aerospace Center (DLR), Hamburg, Germany; 3 Technical University of Munich, TUM Medical Education Center, School of Medicine, Munich, Germany; 4 University of Augsburg, Faculty of Medicine, Deanery, Augsburg, Germany

**Keywords:** clinical reasoning, competence-based assessment, knowledge, strain, teamwork

## Abstract

**Background: **Clinical reasoning refers to a thinking process including medical problem solving and medical decision making skills. Several studies have shown that the clinical reasoning process can be influenced by a number of factors, e.g. context or personality traits, and the results of this thinking process are expressed in case presentation. The aim of this study was to identify factors, which predict the quality of case summary statements as an indicator of clinical reasoning of undergraduate medical students in an assessment simulating the first day of residency.

**Methods: **To investigate factors predicting aspects of clinical reasoning 67 advanced undergraduate medical students participated in the role of a beginning resident in our competence-based assessment, which included a consultation hour, a patient management phase, and a handover. Participants filled out a Post Encounter Form (PEF) to document their case summary statements and other aspects of clinical reasoning. After each phase, they filled out the Strain Perception Questionnaire (STRAIPER) to measure their situation dependent mental strain. To assess medical knowledge the participants completed a 100 questions multiple choice test. To measure stress resistance, adherence to procedures, and teamwork students took part in the Group Assessment of Performance (GAP) test for flight school applicants. These factors were included in a multiple linear regression analysis.

**Results: **Medical knowledge and teamwork predicted the quality of case summary statements as an indicator of clinical reasoning of undergraduate medical students and explained approximately 20.3% of the variance. Neither age, gender, undergraduate curriculum, academic advancement nor high school grade point average of the medical students of our sample had an effect on their clinical reasoning skills.

**Conclusion:** The quality of case summary statements as an indicator of clinical reasoning can be predicted in undergraduate medical students by their medical knowledge and teamwork. Students should be supported in developing abilities to work in a team and to acquire long term knowledge for good case summary statements as an important aspect of clinical reasoning.

## Zusammenfassung

**Hintergrund: **Das klinische Argumentieren bezieht sich auf Denkprozesse, die sowohl medizinisches Problemlösen und als auch eine medizinische Entscheidungskompetenz beinhalten. In mehreren Studien wurde gezeigt, dass der klinische Argumentationsprozess durch eine Reihe von Faktoren beeinflusst wird, z.B. durch kontextuelle Faktoren oder Persönlichkeitsmerkmale, und die Ergebnisse dieses Denkprozesses werden in der Fallpräsentationen ausgedrückt. Ziel dieser Studie war es, die Qualität von Fallzusammenfassungen als Indikator des klinischen Argumentierens bei Medizinstudierenden an einem simulierten ersten Arbeitstag in der ärztlichen Weiterbildung zu ermitteln.

**Methoden:** Um zu untersuchen, welche Faktoren Aspekte des klinischen Argumentierens vorhersagen, nahmen 67 fortgeschrittene Medizinstudierende in der Rolle eines Arztes oder Ärztin in Weiterbildung an unserer kompentenzbasierten Prüfung teil, die eine Sprechstunde, eine Patientenmanagementphase und ein Übergabegespräch umfasste. Die Studierenden füllten ein Freitextformular (PEF – Post Encounter Form) aus, um ihre Fallzusammenfassungen und andere Aspekte des klinischen Argumentierens zu dokumentieren. Im Anschluss an jede Phase machten sie im Fragebogen zum aktuellen Beanspruchungsempfinden (STRAIPER) Angaben zu ihrer wahrgenommenen Befindlichkeit. Um das medizinische Fachwissen zu erfassen, absolvierten sie einen Multiple Choice Test mit 100 Fragen. Um Belastbarkeit, Regelorientierung und Zusammenarbeit zu messen, nahmen die Studierenden am Group Assessment of Performance (GAP) Test für Flugschulanwärterinnen und -anwärter teil. Diese Faktoren waren Grundlage einer multiplen linearen Regressionsanalyse.

**Ergebnisse: **Medizinisches Fachwissen und Zusammenarbeit prognostizieren die Qualität von Fallzusammenfassungen als Indikator des klinischen Argumentierens von Medizinstudierenden und erklären etwa 20,3% der Varianz. Weder Alter, Geschlecht, Art des Curriculums, Studienfortschritt noch die Abiturdurchschnittsnote der Studierenden hatten einen Einfluss auf die Fähigkeit des klinischen Argumentierens.

**Schlussfolgerung: **Die Qualität von Fallzusammenfassungen als Indikator des klinischen Argumentierens von Medizinstudierenden kann durch medizinisches Fachwissen und Zusammenarbeit vorhergesagt werden. Studierende sollten dabei unterstützt werden, Teamfähigkeit zu entwickeln und langfristiges Fachwissen für gute Fallzusammenfassungen als wichtigen Aspekt des klinischen Argumentierens zu erwerben.

## Introduction

Clinical reasoning is the cognitive process physicians use to investigate patients’ problems based on the information gathered from history, physical examination, and sometimes additional test results to make a diagnosis [1]. The different aspects and skills of clinical reasoning need to be developed during undergraduate medical training, e.g. in seminars [2], and should be refined during postgraduate medical education [3]. The context of clinical encounters has an additional influence on the way clinical reasoning is executed [4]. Within the diagnostic process, different levels of expertise are associated with different approaches of reasoning [5] and include the intuitive and the hypothetic deductive way for making decisions [6]. 

Case presentations are used as a teaching format which includes and summarizes aspects of clinical reasoning [7]. Tools have been developed to teach and assess indicators of clinical reasoning during oral or written case presentations which constitute the result of the diagnostic thinking process [8], [9]. Presenting patients in front of others, e.g. during morning report, is a necessary requirement to summarize and share the clinical reasoning process with colleagues but can be a stressful experience [10]. Furthermore, knowledge plays an important role in how new information is processed and reflect upon in the thinking process [11]. Additionally, contextual factors such as emotional reactions can impact on medical students’ clinical reasoning [12]. Signs of strain perception, which often occur with the start of medical students’ clinical rotations [13], might influence clinical reasoning negatively, because if demanding work becomes straining, its outcome can be reduced [14]. Therefore, stress resistance is a desired characteristic in medical students, which is sometimes included in multiple mini interviews for medical school admission [15].

In addition to contextual factors, personality specific factors can also influence academic performance [16]. During Objective Structured Clinical Examinations (OSCEs), for instance, advanced medical students were observed to show high scores in procedural skills, which require a certain amount of adherence to procedures [17]. Good teamwork also plays an indispensable role in in the daily routine on the ward [18] and especially the distribution and exchange of information within a team are crucial for the clinical reasoning process [19]. In medical education, real or virtual simulations have been shown to elucidate aspects of clinical reasoning and provide insights into developing clinical reasoning teaching or assessment formats [20], [21]. Aspects of clinical reasoning or the results of the clinical reasoning process and its factors of influence can be assessed in three different ways, namely by non-workplace-based assessment, by workplace-based assessment or by assessment in simulated clinical environments [22]. Taken together, further studies are needed to explore factors of potential influence on the clinical reasoning process resulting in possible differences of case presentation quality.

In this study we examined, which factors can predict the quality of case summary statements in undergraduate medical students in an assessment simulating the first day of residency. We focus particularly on case presentation as the result of the clinical reasoning process. Based on the current literature we hypothesize that medical knowledge, perceived strain, stress resistance, teamwork, and adherence to procedures are primary predictors for the quality of case summary statements as an important indicator of clinical reasoning in our simulated medical context. 

## Methods

### Procedure

The study took place in July 2017. The evaluation of the quality of case summary statements as an indicator of clinical reasoning was part of a 360 degree competence assessment of undergraduate medical students in the role of beginning residents in a simulated first workday of residency [23]. This assessment was based on the selected competences relevant for beginning residents [24] and it represented a maximal simulation of a clinical environment. Every participant held an individual consultation hour with five simulated patients. The patient cases could not be solved by pattern recognition and included a woman with atrial fibrillation, a man with granulomatous polyangiitis, a woman with perforated sigma diverticulitis, a man with covered perforated infrarenal aortic aneurysm and an immunosuppressed woman with herpes zoster [23].This was followed by a management phase (2.5 hours), where the participants could organize their patients’ next diagnostic steps and interacted with other health care personnel. Eventually, the participants handed their patients over to a resident in 30 minutes.

#### Participants

The participants of our study were 67 advanced undergraduate medical students (semester 10 to 12) from three medical schools with different curricula of 12 semesters who had volunteered to participate. They were selected on first-come first-served terms and received a 25 Euro book voucher after completion of the assessment. Data from five had to be excluded, because their data sets were incomplete with respect to the summary statements. Data from 62 participants (n=32 from the University of Hamburg, n=6 from the University of Oldenburg, n=24 from the Technical University Munich) were included in our analysis. The mean age of the 35 female and 27 male students was 26.1±2.2 years. 

#### Instruments

During the 360-degree assessment, participants filled out one free text Post Encounter Form (PEF) [9] per patient during the management phase of the assessment. This form provides a scoring system including the items summary statement, list of problems, list of differential diagnoses, most likely diagnosis, and supporting data for most likely diagnosis. In our study, we focused only on the summary statement as an important indicator of clinical reasoning [2], which provided the basis for the patient handover. The summary statements were assessed as one aspect of clinical reasoning by an experienced physician with respect to two aspects: the adequacy of the presentation of the summary with respect to medical content (rating scale 1) and with respect to the use of proper medical terminology for symptoms and findings (rating scale 2). Rating scale 1 included the following 5 point Likert scale [9]: 1=“Unable to summarize”, 2=“Poor/ inadequate summary”, 3=“Adequate summary”, 4=“Well summarized, recognizes key details”, 5=“Outstanding summary, demonstrates understanding”. Rating scale 2, also a 5 point Likert scale, consists of the following items: 1=“Uses lay terms or patient’s word”, 2=“Incorrect use of medical language”, 3=“Correctly uses some medical terminology”, 4=“Frequently and correctly uses medical terminology”, 5=“Advanced fluency in medical terminology, eloquent and concise” [9]. We calculated a score of the combined results from scale 1 and 2 per participant [9], resembling the mean of the scores for the five patients per participant: 1=“Participant's performance is below expectations”, 2=“Participant's performance is partly in line with expectations”, 3=“Participant's performance is in line with expectations”, 4=“Participant's performance exceeds expectations”, and 5=“Participant's achievements exceed expectations by far”. The internal consistency (Cronbach’s alpha) of the assessment of the case summary statements of the PEF based on the average of these two items in our study sample was .57. 

After each of the three phases of the 360 degree assessment, we measured students’ perceived strain, an aversive response produced in the individual by potentially harmful exposure, which can be expressed as stress in its strongest occurrence [25]. The students filled out the Strain Perception Questionnaire (STRAIPER) [26], which is based on the QCD (Short questionnaire on current disposition) by Müller and Basler [27] and includes the following six bi polar items: tension (calm versus tense), doubt (confident versus doubtful), concern (unconcerned versus worried), agitation (unwound versus agitated), discomfort (comfortable versus uncomfortable), and apprehensiveness (relaxed versus apprehensive). The questionnaire serves the quantification of situation-dependent subjective mental strain. The scale includes a 6-point Likert scale (1=very low level to 6=very high level) for every questionnaire item. In the current analysis, only the STRAIPER measurements after the consultation hour were included as means of the six items. The Cronbach’s alpha of the STRAIPER is .78.

All participants completed a case based multiple choice test with 100 questions (and a maximum of 100 points) to assess their medical knowledge one week before the assessment. This knowledge test was compiled from 1000 freely available United States Medical Licensing Examination Step 2 type items [28]. The selection process of the questions is described elsewhere [29].

Furthermore, participants of the 360 degree assessment additionally participated one day later in part of the Group Assessment of Performance (GAP) test [30] used for testing of flight school applicants [31]. It contains a validated 1.5 hours computerized team task to evaluate social and interactive skills. The following competences were assessed: stress resistance (SR), adherence to procedures (AP), and teamwork (TW). Stress resistance is defined as maintaining effective performance, control, and goal orientation under pressure or adversity. Stress resistance includes also the absence of physiological symptoms (vegetative, motoric or verbal). The competence of adherence to procedures is defined by knowledge and disciplined and correct application of rules. Teamwork is characterized by active and constructive cooperation in the group process as well as by asking for ideas and perspectives of others. A comprehensive description of these competences and their facets was given earlier [31]. The observation of the participants was carried out by two DLR aviation psychologist with more than 15 years and 2000 cases of experience in behavioral observation. The observers used a set of empirically derived behaviour checklists [26] to assess each competence on a 6-point Likert scale (1: very low occurrence to 6: very high occurrence). The interrater reliabilities for the DLR pilot assessment center using the GAP-behaviour-observation-procedure are: SR=.82, AP=.75, and TW=.88 [32]. 

#### Statistical analysis

The statistical calculations were performed with SPSS Statistics (version 23) and included a multiple linear regression analysis using a regression model with the following predictors: Medical knowledge, perceived strain, stress resistance, adherence to procedures, and teamwork as well as aspects of clinical reasoning as dependent variable. The significance was set on a *p*-value=.05. All requirements for our linear regression model were fulfilled. Our date showed additivity and linearity, independence of the residuals, variance in all predictors, normally distributed residuals, as well as no multicollinearity. 

## Results

The mean of the quality of our participants’ summary statements as an indicator of clinical reasoning was 2.78 (±.58), whereby 5 was the highest score. On average, they reached 73.3 (±9.1) of 100 possible points in the medical knowledge multiple choice test. On a 6 point scale ranging from 1=“very low” to 6=“very high”, they showed a perceived strain of 3.87 (±.79), a stress resistance score of 4.11 (±.71), an adherence to procedures score of 5.51 (±.63), and a teamwork score of 3.49 (±.83).

**Figure 1:**
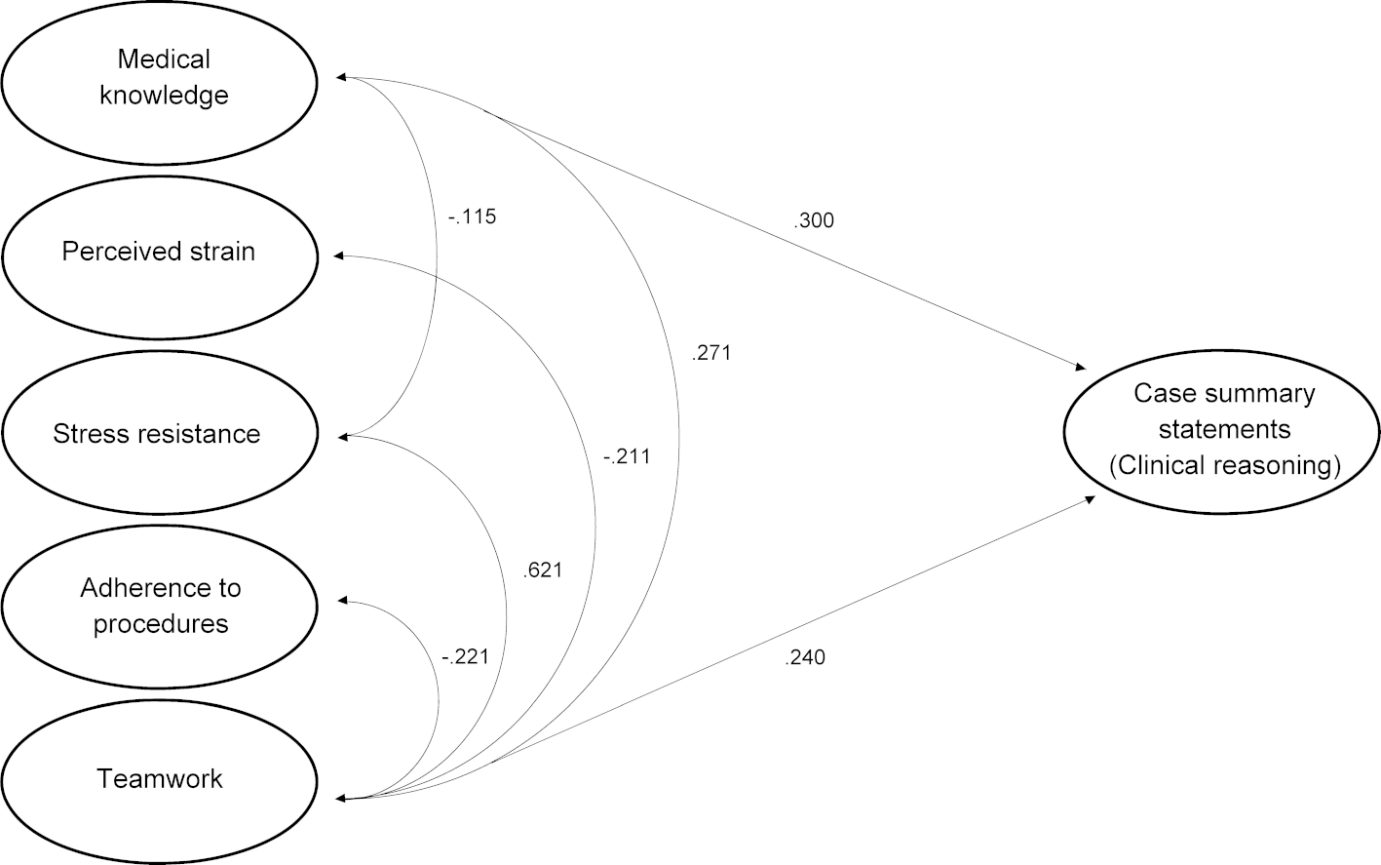
Regression model including correlations between predictors and dependent variable

Two of the predictors, i.e. medical knowledge (10.3%) and teamwork (10.0%), explained in combination a significant portion (20.3%) of the variance of aspects of clinical reasoning (R2=.203, F(5, 62)=2.844, p=.023), shown in table 1 (Tab. 1). Medical knowledge could predict clinical reasoning aspects (β=.372, t(62)=2.788, p=.007) as well as teamwork (β=.401, t(62)=2.521, p=.015). The intercorrelation of all variables of the regression model is shown in table 2 (Tab. 2). There are significant correlations between teamwork and every predictor in our model, whereas perceived strain and adherence to procedures correlate with no other variable. The regression model was controlled by age, gender, undergraduate curriculum, academic advancement, and high school grade point average, which had no effect on clinical reasoning. An overview of the regression model with all predictors (medical knowledge, perceived strain, stress resistance, adherence to procedures, teamwork) is provided in figure 1 (Fig. 1).

## Discussion

In our assessment, the quality of case summary statements as an indicator of clinical reasoning is predicted by two factors. Medical knowledge explained 10.3% of the variance of the quality of the case summary statements. It is the basis of clinical reasoning, and decision making cannot begin without the necessary knowledge on medical subjects [33]. In another study, postgraduate students with better knowledge of basic clinical care also showed better clinical reasoning skills in an exam [34]. The more medical students’ knowledge increased over the time in a progress test at a medical school with a problem-based curriculum, the greater certain indicators of their clinical reasoning became [35]. However, even though third year postgraduate medical students have greater medical knowledge, they were observed to commit similar heuristic errors in clinical reasoning to those of residents in their first year [36]. Proficiency in medical knowledge and clinical reasoning, particularly in presenting cases, is linked, since specialized vocabulary is acquired while students gain experience and improve their understanding of diseases [37]. 

The factor teamwork explained 10.0% of the variance in quality of the case summary statements as an indicator of clinical reasoning in our study. In a human patient simulation for pharmaceutical students, clinical judgement and problem-solving skills, which are needed for clinical reasoning, were improved in combination with teamwork while solving the cases successfully [38]. Dental students who participated in a problem based learning course also reported an increase in their teamwork and problem solving skills [39]. Teamwork has also been shown to facilitate the development of creative solutions to challenging problems [40]. Simulation based learning in teams during undergraduate medical education can also be fostered by using collaboration scripts [41]. Even though no explicit teamwork task for clinical reasoning was implemented in our 360 degree assessment, participants could discuss their thoughts on the management of the patients with their supervisor or other health care personnel and could request laboratory and radiology tests before writing their case summary statements.

Contextual factors like the emotional reaction of students (e.g. to patients’ conditions or behavior) and the physician patient relationship were found to have an impact on clinical reasoning [12]. In our study, students‘ perceived strain did not predict the quality of case summary statements as an indicator of clinical reasoning, which could be due to the fact that the students perceived moderate strain, but not actually stress during the simulated work day. However, no differences with respect to diagnostic accuracy and clinical reasoning arguments between stressed and less stressed students were found elsewhere [42]. 

During the GAP test students showed the highest scores for adherence to procedures compared to the scores for the other competences [30]. However, adherence to procedures did not predict clinical reasoning. Apparently, the clinical reasoning process, reflected by the quality of case summary statements, requires additional skills than just carefully following the rules. It refers to a thinking process, which includes medical problem solving and medical decision making skills [33]. Furthermore, it requires the ability to switch from intuitive to analytic thing to make correct diagnoses when patient cases are complex [43]. 

A strength of our study is the fact that students from medical schools with different undergraduate curricula and with different academic advancement participated. This enabled us to control for these factors in our analysis. A weakness of our study is that the summary statements were assessed by just one experienced physician. However, she has been teaching clinical reasoning for many years [2] and was involved in the design and operationalization of the 360 degree assessment [23]. Furthermore, good interrater reliability has been shown for the instrument [44] and the original instrument was used by one rater for the assessment of the case summary statements [9]. However, a better approach would have been to evaluate the whole Post Encounter Form and calculate a regression analysis. Unfortunately, only very few of the participating students completed the whole form which would have reduced the sample number to an extent that would have made a regression analysis impossible. Despite the low sample number, we were able to identify significant predictors of clinical reasoning measured with a validated scoring form [9]. On the other hand, the low reliability for the assessment of the case summary statements of the PEF is another limitation of or study. However, with our simulation we created a realistic environment to investigate factors, which can influence aspects of clinical reasoning, supported by the validated GAP-test used for flight school applicants [31].

## Conclusion

Medical knowledge and teamwork predicted the quality of case summary statements as an indicator of clinical reasoning of undergraduate medical students from medical schools with different curricula and with different academic advancement during a simulated first day of residency. Teamwork supports a good quality of case summary statements as an aspect clinical reasoning, which might be due to teamwork involving social sensitivity and exchange of information. Thus, it might be useful to support medical students in developing the ability to work in teams as well as to acquire long term knowledge for improving the quality of their case summary statements as an indicator for clinical reasoning. 

## Study

This study was part of the project ÄKHOM, supported by a grant from the Federal Ministry of Education and Research (BMBF), reference number: 01PK1501A/B/C. The study was performed in accordance with the Declaration of Helsinki and the Ethics Committee of the Chamber of Physicians, Hamburg, confirmed the innocuousness of the study with consented, anonymized, and voluntary participation (PV3649).

## Acknowledgements

We thank all the medical students who participated in this study. 

## Competing interests

The authors declare that they have no competing interests. 

## Figures and Tables

**Table 1:**
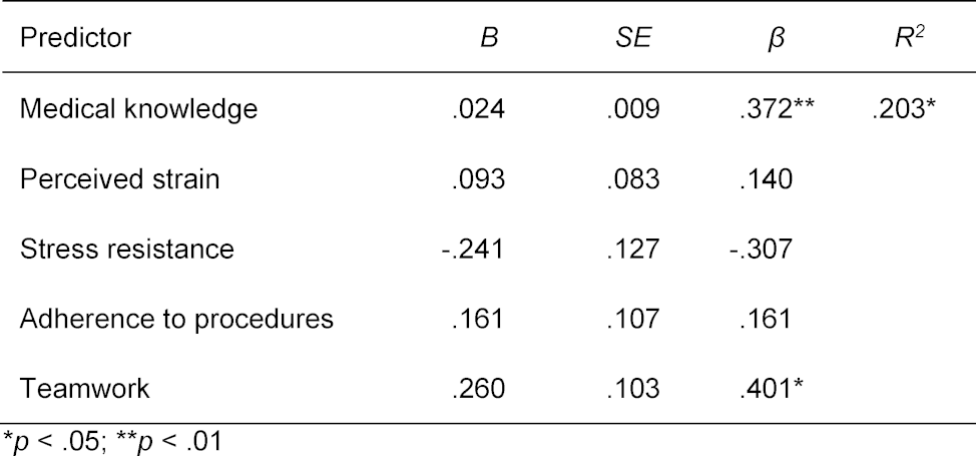
Multiple regression coefficients to predict aspects of clinical reasoning

**Table 2:**
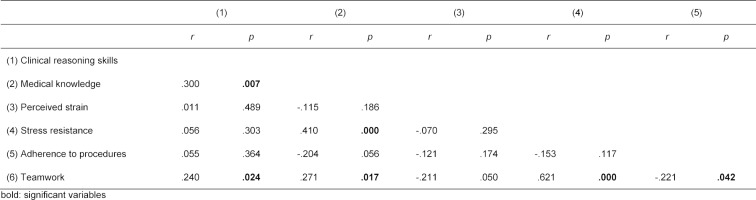
Intercorrelation of all variables of the regression model
